# Psychosocial factors and colleagues’ perceptions of return-to-work opportunities for workers with a psychiatric disorder: a Japanese population-based study

**DOI:** 10.1186/s12199-017-0630-y

**Published:** 2017-04-04

**Authors:** Hisashi Eguchi, Koji Wada, Yoshiyuki Higuchi, Derek R. Smith

**Affiliations:** 1 Department of Public Health, Kitasato University School of Medicine, 1-15-1 Kitasato, Minami-ku, Sagamihara, Kanagawa 252-0374 Japan; 2 Takemi Program in International Health, Department of Global Health and Population, Harvard T.H. Chan School of Public Health, Boston, USA; 3 Bureau of International Health Cooperation, National Center for Global Health and Medicine, Tokyo, Japan; 4 Department of Health and Physical Education, Fukuoka University of Education, Munakata, Japan; 5 College of Public Health, Medical and Veterinary Sciences, James Cook University, Townsville, Australia

**Keywords:** Mental health, Return to work, Psychosocial factors, Socioeconomic factors

## Abstract

**Background:**

This study examined associations between psychosocial factors and the perception that adequate employment opportunities might not be provided for people with limited work capacity due to psychiatric disorders.

**Methods:**

We conducted an online, cross-sectional survey of 3,710 employed individuals aged 20 to 69 years in Japan. Our survey included the Brief Job Stress Questionnaire and investigated participants’ perception of opportunities in their workplace for individuals with a psychiatric disorder returning to work (colleagues’ negative perception) and psychosocial factors (job demand, job control, and workplace social support). Multiple logistic regression analysis was used to evaluate potential associations between psychosocial factors and colleagues’ negative perception.

**Results:**

Colleagues’ negative perception was associated with low workplace social support (middle tertile: Odds Ratio [OR]: 1.26, 95% Confidence Interval [CI]: 1.12–1.40; low tertile: OR 1.45, 95% CI: 1.32–1.58; p for trend <0.01); low levels of job control (middle tertile: OR 1.22, 95% CI: 1.06–1.38; low tertile: OR 1.64, 95% CI: 1.46–1.81; p for trend <0.01); and no previous experience working with a person with a psychiatric disorder (OR 1.74, 95% CI: 1.60–1.88).

**Conclusions:**

Psychosocial factors may affect colleagues’ perceptions of individuals with a psychiatric disorder returning to work in Japan. Greater consideration of psychosocial factors in the workplace may be necessary to facilitate people with a psychiatric disorder successfully returning to work in Japan, as elsewhere.

## Background

Predictors of the successful return to work for individuals with psychiatric disorders have often focused on workplace psychosocial factors as well as individual health conditions [1–4]. Sick leave and withdrawal from the labor market related to mental health has increased the financial burden on society and workplaces through compensation costs and lost productivity [5]. To reduce the economic consequences associated with long-term sick leave and withdrawal from the labor market, a better understanding of the factors that facilitate or complicate return to work for employees with psychiatric diseases is necessary and appropriate [4].

Social support is known to be important for workers, particularly those with a psychiatric disease who are wishing to return to work [1, 3, 6]. Support from colleagues is essential for implementing any necessary measures to reduce work hours, responsibilities, and workloads of employees in this regard. Expectations and actual experiences of the social support available may, however, influence the feelings, thoughts, and behavior of employees with a psychiatric disorder who wish to return to work [4, 7–10]. Despite this fact, research investigating colleague’s perceptions of psychiatric disorders has generally been limited, especially in Japan.

Identifying specific psychosocial factors that may negatively affect colleagues’ perceptions is clearly important as it can facilitate the successful return to work of employees with a psychiatric disorder. The present study aimed, therefore, to examine potential associations between psychosocial factors and colleagues’ negative perceptions in a Japanese working population by means of an internet-based survey.

## Methods

### Participants and survey method

A cross-sectional, online survey was conducted in December 2014 among participants who had previously registered with a Japanese web survey company. In total, 389,874 individuals (excluding those who were self-employed, unemployed, or students) aged 20 to 69 years, without sex or age bias, were invited to participate. The study population comprised individuals who were interested in participating in a survey with a financial incentive for responding, although the actual financial incentives for participation were modest (equivalent to a few US dollars). Participants were selected using a random number generator. The sex ratio was 1:1 and there were an equal number of participants in each 10-year age group (20–29, 30–39, 40–49, 50–59, and 60–69 years). To reflect the non-medical working population in Japan, individuals who were registered as doctors, nurses, and other medical staff were excluded from participation. Recruitment ceased when the number of participants exceeded 3,600, owing to the study’s budgetary constraints.

### Outcome measures (colleagues’ negative perception of return-to-work opportunities)

Similar to a previous study [11], colleagues’ negative perception was determined by participants’ responses to the following question: “Would your workplace climate be able to provide a work opportunity for an employee with limited work capacity owing to symptoms related to psychiatric disorders such as depression and schizophrenia, and the side effects of treatment for those psychiatric disorders?” This particular question was designed to have meaning in Japanese language, considering that organizational climate is known to have a significant effect on the employees’ behaviors and perceptions [12]. A workplace’s organizational climate generally reflects employees’ perceptions of the significance and meaning of their work environment [13], and emerges from the perceptions of workplace members [14]. This question was based on the assumption that a colleagues’ attitude towards a co-worker whose capacity was affected due to a psychiatric disorder (such as schizophrenia or depression), would be influenced by the organizational climate of their workplace. Responses were initially measured on a 4-point scale (1 = definitely yes; 2 = I guess so; 3 = I guess not; 4 = definitely not), and then dichotomized on a 2-point scale: 0 = yes (definitely yes and I guess so); and 1 = no (I guess not and definitely not).

### Psychosocial factors in the workplace

Psychosocial factors in the workplace were defined according to a job demand-control model [15] or a demand-control-support model [16]. Job demand, job control, and workplace social support were evaluated using the Brief Job Stress Questionnaire [17], which consists of 6 items measured on a 4-point scale. Scores range from 6 to 24, with a higher score indicating a greater workload. Job control was measured with three items, with responses on the same 4-point scale; and scores ranging from 3 to 12, with a higher score indicating greater job control and opportunity to participate in workplace decision-making. Both supervisor and coworker support were measured on 3-item scales, with scores ranging from 3 to 12. The sum of the supervisor and coworker support scales (range 6 to 24) indicated total workplace social support, with a higher score suggesting better workplace relationships. The Brief Job Stress Questionnaire scales are known to have acceptable levels of internal consistency, reliability, and factor-based validity [17]; Participants were classified into tertiles (low, middle, and high) according to their psychosocial factors scores.

### Other covariates

Other covariates investigated in this study included sex, age, socioeconomic factors, and prior experience working with a person with a psychiatric disorder. Socioeconomic factors included company size, occupation, educational level, and type of employment contract. Company size was classified into three groups (49 or fewer, 50–299, and 300 or more workers). Occupation was classified as manager, white-collar, or blue-collar; with participants asked if they were a manager, and those who were not managers were asked if they were white- or blue-collar workers. Educational level was divided into three categories (junior high school or high school, technical college or junior college, and university and graduate school). Individual employment contracts were classified as being a regular employee or non-regular employee. Previous experience of working with an employee with a psychiatric disorder was assessed by the question, “Have you ever worked with someone whose work capacity was limited due to symptoms related to psychiatric disorders such as depression and schizophrenia, and the side effects of their treatment?” Response options for this question were yes (0) and no (1).

### Statistical analysis

Chi-square analyses were initially undertaken, with logistic regression used to calculate crude and adjusted odds ratios (ORs) and 95% confidence intervals (CIs) for the associations of psychosocial factors in the workplace and other covariates with the outcome of interest (colleagues' negative perception of return-to-work opportunities). Crude ORs were calculated one by one for all variables. Adjusted ORs were adjusted for sex, age, marital status, educational level, employment contract, occupation, company size, previous experience working with an employee with a psychiatric disorder, job demand, job control, and workplace social support. Given that the study outcome was not rare, we applied Zhang’s formula of adjustment when calculating the results of regression analyses [18]. Our equation was that P0 indicates the incidence of the outcome of interest in the non-exposed group and P1 in the exposed group; OR, odds ratio; and RR, risk ratio: OR = (P1/1-P1)/(P0/1-P0); thus, (P1/P0) = OR/[(1-P0) + (P0*OR)]. Since RR = P1/P0, the corrected RR = OR/[(1-P0) + (P0*OR)]. The Cochran-Armitage Test for Trend [19, 20] was used to determine the statistical significance of trends over an ordinal scale. As the interaction between sex and workplace social support was significant (p for interaction = 0.02), gender-based sensitivity analyses were conducted to confirm the associations between psychosocial factors and colleagues’ negative perception by gender. Statistical analyses were performed using SPSS Version 22.0 (IBM SPSS Statistics for Windows, Version 22.0. Armonk, NY, USA). A two-tailed *p*-value of 0.05 was considered significant, unless otherwise indicated.

### Ethics

The study aims and protocol were approved in 2014 by the Institutional Ethics Committee of the Kitasato University School of Medicine (B14-146). Participants were informed in advance that their participation was strictly voluntary and that all information provided would remain confidential. Those who consented to participate were able to access a designated website on verification of their personal information, after which they could complete the survey online. Participants had the option not to respond to any part of the questionnaire, and could discontinue participation at any point.

## Results

In total, 3,710 individuals (1,855 males and 1,855 females) participated in the study and their background characteristics are shown in Table 1. Slightly more than half the participants (52.2%) reported that their workplace climate could not provide a work opportunity for an employee with limited work capacity due to a psychiatric disorder and/or treatment side effects. One-fifth of participants reported a previous experience of having worked with a person with a psychiatric disorder.

**Table 1:** Participant characteristics (*n* = 3,710)

		Number	(%)
Colleague’s perceptions			
	Having a negative perception	1936	(52.2)
	Not having a negative perception	1774	(47.8)
Gender			
	Male	1855	(50.0)
	Female	1855	(50.0)
Age range (years)			
	20-29	742	(20.0)
	30-39	742	(20.0)
	40-49	742	(20.0)
	50-59	742	(20.0)
	60-69	742	(20.0)
Workplace social support score			
	High (18–24)	1151	(31.0)
	Middle (15–17)	950	(25.6)
	Low (6–14)	1609	(43.4)
Job control score			
	High (10–12)	521	(14.0)
	Middle (8–9)	1663	(44.8)
	Low (3–7)	1526	(41.1)
Psychological demand score			
	Low (6–14)	1317	(35.5)
	Middle (15–17)	1228	(33.1)
	High (18–24)	1165	(31.4)
Previous experience with colleagues with a psychiatric disorder			
	Yes	772	(20.8)
	No	2938	(79.2)
Company size (number of employees)			
	300+	1000	(27.0)
	50 to <300	1415	(38.1)
	0 to <50	1295	(34.9)
Occupation			
	Manager	341	(9.2)
	White collar employee	2557	(68.9)
	Blue collar employee	812	(21.9)
Educational level			
	University and/or graduate school	1674	(45.1)
	Technical college or junior college	904	(24.4)
	Junior high school or high school	1132	(30.5)
Employment contract			
	Regular	1980	(53.4)
	Non-regular	1730	(46.6)

Table 2 describes statistical associations between psychosocial factors and colleagues’ negative perception. A relatively high proportion of participants who reported they had low workplace social support, low levels of job control, no previous experience working with an employee with a psychiatric disorder, worked in a small company, were blue-collar workers, or had low educational level reported that their workplace climate could not provide a working opportunity for an employee with limited work capacity owing to symptoms related to a psychiatric disorder and the side effects of treatment for those psychiatric disorders.

**Table 2:** Associations between psychosocial and socioeconomic factors and colleagues’ negative perception (*n* =3,710)

		Organizational climate				
		Having a negative perception		Not having a negative perception		p^a^
		*n* =1936	(%)	*n* =1774	(%)	
Gender						
	Male	883	(47.6)	972	(52.4)	<0.01
	Female	1053	(56.8)	802	(43.2)	
Age range (years)						
	20–29	375	(50.5)	367	(49.5)	<0.01
	30–39	353	(47.6)	389	(52.4)	
	40–49	365	(49.2)	377	(50.8)	
	50–59	407	(54.9)	335	(45.1)	
	60–69	436	(58.8)	306	(41.2)	
Workplace social support score						
	High (18–24)	483	(42.0)	668	(58.0)	<0.01
	Middle (15–17)	491	(51.7)	459	(48.3)	
	Low (6–14)	952	(59.8)	647	(40.2)	
Job control score						
	High (10–12)	202	(38.8)	319	(61.2)	<0.01
	Middle (8–9)	788	(47.4)	875	(52.6)	
	Low (3–7)	946	(62.0)	580	(38.0)	
Job demand score						
	Low (6–14)	709	(53.8)	608	(46.2)	0.16
	Middle (15–17)	644	(52.4)	564	(47.6)	
	High (18–24)	583	(50.0)	582	(50.0)	
Previous experience with colleagues with a psychiatric disorder						
	Yes	250	(32.4)	522	(67.6)	<0.01
	No	1686	(57.4)	1252	(42.6)	
Company size (number of employees)						
	300+	410	(41.0)	590	(59.0)	<0.01
	50–299	737	(52.1)	678	(47.9)	
	0–49	789	(60.9)	506	(39.1)	
Occupation						
	Manager	135	(39.6)	206	(60.4)	<0.01
	White-collar employee	1319	(51.6)	1238	(48.4)	
	Blue-collar employee	482	(59.4)	330	(40.6)	
Educational level						
	University and/or graduate school	767	(45.8)	907	(54.2)	<0.01
	Technical college or junior college	500	(55.3)	404	(44.7)	
	Junior high school or high school	669	(59.1)	463	(40.9)	
Employment contract						
	Regular	945	(47.7)	1035	(52.3)	<0.01
	Non-regular	991	(57.3)	739	(42.7)	

^a^Chi-square

Table 3 describes the results of the multiple logistic regression analyses of associations between psychosocial factors and colleagues’ negative perceptions. Colleagues’ negative perceptions were associated with lower workplace social support when compared with high workplace social support (middle tertile: Odds Ratio [OR]: 1.26, 95% Confidence Interval [CI]: 1.12–1.40; low tertile: OR: 1.45, 95% CI: 1.32–1.58; p for trend < 0.01); lower job control when compared with high job control (middle tertile: OR: 1.22, 95% CI: 1.06–1.38; low tertile: OR: 1.64, 95% CI: 1.46–1.81; p for trend < 0.01); not having experience of working with an employee with a psychiatric disorder when compared with having previous experience (OR: 1.74, 95% CI: 1.60–1.88); smaller company size when compared with companies of more than 300 employees (50–299: OR: 1.25, 95% CI: 1.12–1.38; <50 employees: OR: 1.51, 95% CI: 1.37–1.66; p for trend <0.01); white- or blue-collar occupations when compared with managers (white-collar: OR: 1.14, 95% CI: 0.95–1.34; blue-collar: OR: 1.20, 95% CI: 0.99–1.43); and, low educational level (technical college or junior college: OR: 1.09, 95% CI: 0.97–1.22; junior high school or high school: OR: 1.16, 95% CI: 1.04–1.30; p for trend <0.01). The participant’s employment contract was not significantly associated with colleagues’ negative perception.

**Table 3:** Multiple logistic regression analysis of associations between psychosocial factors and colleagues’ negative perception (*n* =3,710)

		Crude OR (95% CI)		Adjusted OR (95% CI)^a^	
Gender					
	Male	1.00		1.00	
	Female	1.27	(1.17–1.37)	1.19	(1.08–1.31)
Age range (years)					
	20–29	1.00		1.00	
	30–39	0.92	(0.79–1.06)	0.95	(0.81–1.10)
	40–49	0.97	(0.83–1.10)	0.94	(0.81–1.10)
	50–59	1.12	(0.98–1.28)	1.11	(0.96–1.28)
	60–69	1.24	(1.09–1.40)	1.22	(1.05–1.40)
	p for trend	*p* < 0.01		*p* < 0.01	
Workplace social support score					
	High	1.00		1.00	
	Middle	1.29	(1.16–1.42)	1.26	(1.12–1.40)
	Low	1.54	(1.42–1.67)	1.45	(1.32–1.58)
	p for trend	*p* < 0.01		*p* < 0.01	
Job control score					
	High	1.00		1.00	
	Middle	1.25	(1.10–1.41)	1.22	(1.06–1.38)
	Low	1.72	(1.56–1.88)	1.64	(1.46–1.81)
	p for trend	*p* < 0.01		*p* < 0.01	
Job demand score					
	Low	1.00		1.00	
	Middle	0.97	(0.86–1.07)	1.01	(0.90–1.13)
	High	0.90	(0.80–1.01)	1.07	(0.95–1.20)
	p for trend	*p* = 0.06		*p* = 0.27	
Previous experience with colleagues with a psychiatric disorder					
	Yes	1.00		1.00	
	No	1.79	(1.66–1.92)	1.74	(1.60–1.88)
Company size (number of employees)					
	300+	1.00		1.00	
	50–299	1.33	(1.20–1.46)	1.25	(1.12–1.38)
	1–49	1.61	(1.48–1.75)	1.51	(1.37–1.66)
	p for trend	*p* < 0.01		*p* < 0.01	
Occupation					
	Manager	1.00		1.00	
	White-collar employee	1.36	(1.18–1.40)	1.14	(0.95–1.34)
	Blue-collar employee	1.61	(1.40–1.81)	1.20	(0.99–1.43)
Educational level					
	University and/or graduate school	1.00		1.00	
	Technical college or junior college	1.28	(1.15–1.40)	1.09	(0.97–1.22)
	Junior high school or high school	1.40	(1.28–1.52)	1.16	(1.04–1.30)
	p for trend	*p* < 0.01		*p* < 0.01	
Employment contract					
	Regular	1.00		1.00	
	Non-regular	1.28	(1.18–1.38)	1.01	(0.91–1.13)

^a^Adjusted for sex, age, marital status, educational level, employment contract, occupation, company size, previous experience working with an employee with a psychiatric disorder, job demand, job control, and workplace social support

A sensitivity analysis conducted separately by gender found similar results to when sex was entered into the model as a variable, and confirmed the linear associations between colleagues’ negative perceptions and age, psychosocial factors, experience working with an employee with a psychiatric disorder, company size, and educational level.

Cronbach’s alpha coefficients were 0.84 for job demand, 0.75 for job control, 0.84 for supervisor support, 0.83 for coworker support, and 0.87 for total workplace social support.

## Discussion

This study investigated associations between psychosocial factors and negative perceptions of colleagues in Japan’s working-age population. Several potential determinants of colleagues’ negative perception were identified, with around half of all participants reporting a negative perception of employment opportunities for a potential colleague with limited work capacity due to a psychiatric disorder or related treatment. Being female, older age, having lower levels of workplace social support and job control, not having previous experience working with an employee with a psychiatric disorder, smaller company size, and lower educational level were independently associated with colleagues’ negative perceptions.

A statistically significant association was demonstrated between adverse workplace-based psychosocial factors (low workplace support and low job control) and colleagues’ negative attitudes toward a coworker with a psychiatric disease. Previous studies have shown that psychosocial factors including workplace social support and job control are important contributors to the organizational climate in Japanese workplaces [12]. Similarly, adverse working environments (low workplace social support and low job control) may also be inversely associated with successful return to work of employees with a psychiatric disorder [21]. Supervisor and coworker support are known to be important determinates of workplace mental health [22] and have also been demonstrated as a predictor for the successful return to work of employees with a psychiatric disorder [1, 3, 6]. Moreover, adverse job characteristics may lead to a general reduction in the helping of others in the workplace [23]; while suboptimal workplace-based social support may also limit interpersonal helping behavior [24]. Some research suggests that employees who do not feel well-supported by their organizations tend to reciprocate by showing less engagement in interpersonal helping behavior, when compared with those who report higher levels of organizational support [25–28]. Colleagues’ attitudes toward coworkers with a psychiatric disorder may mediate the association between psychosocial factors and successful return to work.

Given these facts, it is reasonable to hypothesize that age reflects an individual’s historical background, and therefore, may play an important contextual role in their reaction to colleagues experiencing a psychiatric disorder. In this study, we found that older age was associated with more negative attitudes towards (hypothetical) psychiatric disorders in the workplace. This finding was consistent with previous studies that reported an association between older age and negative attitudes toward psychiatric disorders [29–32]. After the guidelines for workplace mental health were promulgated in Japan during the year 2000, it is generally believed that workplace activities to reduce stigma and discrimination towards people with psychiatric disorders have been relatively effective in this country [33]. As a result, younger people may have a more positive impression of colleagues with a psychiatric disorder than their older colleagues. As such, it would be useful if the attitudes of these groups are explored in future research, to help develop a greater understanding of how negative attitudes could be more effectively addressed in the less positive sub-groups.

Stigma toward psychiatric disorders in the workplace may comprise an obstacle to a successful return to work [34–36]. Corrigan and colleagues, for example, previously identified three strategies for challenging stigmatizing attitudes and prejudice towards psychiatric disorders, as follows: protest, education, and contact [37]. “Contact” reasons that the less contact an individual has with someone who has experienced psychiatric disorders, the more stigmatizing they will be, although the relationship is correlational; that is it may equally be the case that those who hold less stigmatizing attitudes towards psychiatric disorders are happier to have contact with individuals experiencing psychiatric disorders in this regard. In the workplace, previous experience of working with someone with a psychiatric disorder might reduce the stigmatizing negative perception of a future colleague, similarly encumbered.

In Japan, it is reasonable to assume that small and medium-sized companies may have insufficient human resources for mental health activities when compared with large companies. For example, government statistics suggest that when compared with 100% of large companies, less than 60% of small and medium-sized companies were able to conduct preventive activities to address mental health issues [38]. In addition, another Japanese study found that smaller companies may be less likely to have flexible working systems such as a half-day working hours, a dedicated return to work system, or paid sick leave to accommodate attending regular hospital visits; when compared to larger companies [39]. It is important to note that Japanese companies with 50 or more employees are required by the Occupational Health and Safety Law to employ an occupational health physician. Therefore, this lack of physician supervision may be one reason why workers in smaller Japanese companies are more likely to have negative perceptions of individuals with mental health issues in the workplace.

A limitation of the present study was firstly that the study population was recruited through a web-based survey company, and it might be expected that individuals who can access the internet can also seek information more easily and might take healthy behaviors more seriously [40]. Our results may therefore not be completely generalizable to those without internet access, or to other countries and settings. Secondly, the present study was also cross-sectional, meaning that no causal relationships could be determined. To clarify potential causal relationships between psychosocial factors and colleagues’ negative perception, a further interventional study should be conducted. Thirdly, regarding "previous experience of working with an employee with a psychiatric disorder," we could not confirm the actual medical diagnosis of someone who participants judged that their work capacity was limited due to symptoms related to psychiatric disorders such as depression and schizophrenia and the side effects of their treatment. Participants in the current study might, therefore, have misjudged the conditions of their co-workers. Fourth, we did not specify the symptoms in the questionnaire, and we did not ask the participants how they knew someone had symptoms related to a psychiatric disorder or the side effects of its treatment. The interpretations of “symptoms” may, therefore, vary from participant to participant. Finally, while the present study investigated colleagues’ negative perception, participants were not followed up, meaning that we were unable to determine any subsequent changes in perceptions. This may be a relevant consideration for future investigations.

## Conclusions

Overall, our findings suggest that psychosocial factors may affect colleagues’ perceptions of individuals with a psychiatric disorder returning to work in Japan. Greater consideration of psychosocial factors in the workplace may be necessary to facilitate people with a psychiatric disorder successfully returning to work in Japan, as elsewhere.

## References

[1] De Vries G, Koeter M, Nabitz U, Hees H, Schene A (2012). Return to work after sick leave due to depression; a conceptual analysis based on perspectives of patients, supervisors and occupational physicians. J Affect Disord.

[2] Blank L, Peters J, Pickvance S, Wilford J, MacDonald E (2008). A systematic review of the factors which predict return to work for people suffering episodes of poor mental health. J Occup Rehabil.

[3] Lemieux P, Durand M-J, Hong Q (2011). Supervisors’ perception of the factors influencing the return to work of workers with common mental disorders. J Occup Rehabil.

[4] Andersen MF, Nielsen KM, Brinkmann S (2012). Meta-synthesis of qualitative research on return to work among employees with common mental disorders. Scand J Work Environ Health.

[5] Japan Health Insurance Association The survey on cash sickness beneficiaries 2014. Japan Health Insurance Association. 2015. https://www.kyoukaikenpo.or.jp/~/media/Files/honbu/cat740/2707/270724/270724012.pdf. Accessed 25 Aug 2016 (in Japanese).

[6] Josephson M, Heijbel B, Voss M, Alfredsson L, Vingård E (2008). Influence of self-reported work conditions and health on full, partial and no return to work after long-term sickness absence. Scand J Work Environ Health.

[7] Verdonk P, de Rijk A, Klinge I, de Vries A (2008). Sickness absence as an interactive process: Gendered experiences of young, highly educated women with mental health problems. Patient Educ Couns.

[8] Saint-Arnaud L, Saint-Jean M, Damasse J (2006). Towards an enhanced understanding of factors involved in the return-to-work process of employees absent due to mental health problems. Can J Commun Ment Health.

[9] Noordik E, Nieuwenhuijsen K, Varekamp I, Van der Klink JJ, J van Dijk F (2011). Exploring the return-to-work process for workers partially returned to work and partially on long-term sick leave due to common mental disorders: a qualitative study. Disabil Rehabil.

[10] Hillborg H, Svensson T, Danermark B (2010). Towards a working life? Experiences in a rehabilitation process for people with psychiatric disabilities. Scand J Occup Ther.

[11] Eguchi H, Wada K, Higuchi Y, Smith DR. Co-worker perceptions of return-to-work opportunities for Japanese cancer survivors. 2017; 26:309–15. doi:10.1002/pon.4130. Epub 2016.4.13. 10.1002/pon.4130 27072898

[12] Smith DR, Muto T, Sairenchi T (2011). Examining the dimensions of hospital safety climate and psychosocial risk factors among Japanese nurses. J Transcult Nurs.

[13] Glisson C, James LR (2002). The cross‐level effects of culture and climate in human service teams. J Organ Behav.

[14] Klein KJ, Kozlowski SW (2000). Multilevel theory, research, and methods in organizations: Foundations, extensions, and new directions.

[15] Karasek R, Baker D, Marxer F, Ahlbom A, Theorell T (1981). Job decision latitude, job demands, and cardiovascular disease: a prospective study of Swedish men. Am J Public Health.

[16] Johnson JV, Hall EM (1988). Job strain, work place social support, and cardiovascular disease: a cross-sectional study of a random sample of the Swedish working population. Am J Public Health.

[17] Shimomitsu T, Haratani T, Ohno Y (2000). Development of the Brief Job Stress Questionnaire mainly used for assessment of the individuals: Tokyo, Japan The Ministry of Labour, the 1999 report.

[18] Zhang J, Yu KF (1998). What's the relative risk? A method of correcting the odds ratio in cohort studies of common outcomes. JAMA.

[19] Cochran WG (1954). Some Methods for Strengthening the Common *χ*^2^ Tests. Biometrics.

[20] Armitage P (1955). Tests for Linear Trends in Proportions and Frequencies. Biometrics.

[21] Silva-Junior JS, Fischer FM (2014). Long-term sickness absence due to mental disorders is associated with individual features and psychosocial work conditions. PLoS One.

[22] Smith DR, Wei N, Zhang Y-J, Wang R-S (2006). Musculoskeletal complaints and psychosocial risk factors among physicians in mainland China. Int J Ind Ergonom.

[23] Organ DW, Ryan K (1995). A meta-analytic review of attitudinal and dispositional predictors of organizational citizenship behavior. Pers Psychol.

[24] Chiang C-F, Hsieh T-S (2012). The impacts of perceived organizational support and psychological empowerment on job performance: The mediating effects of organizational citizenship behavior. Int J Hosp Manage.

[25] Wayne SJ, Shore LM, Liden RC (1997). Perceived organizational support and leader-member exchange: A social exchange perspective. Acad Manag J.

[26] Shore LM, Wayne SJ (1993). Commitment and employee behavior: comparison of affective commitment and continuance commitment with perceived organizational support. J Appl Psychol.

[27] Eisenberger R, Fasolo P, Davis-LaMastro V (1990). Perceived organizational support and employee diligence, commitment, and innovation. J Appl Psychol.

[28] Gouldner AW (1960). The norm of reciprocity: A preliminary statement. Am Sociol Rev.

[29] Coppens E, Van Audenhove C, Scheerder G (2013). Public attitudes toward depression and help-seeking in four European countries baseline survey prior to the OSPI-Europe intervention. J Affect Disord.

[30] Aromaa E, Tolvanen A, Tuulari J, Wahlbeck K (2011). Predictors of stigmatizing attitudes towards people with mental disorders in a general population in Finland. Nord J Psychiatry.

[31] Griffiths KM, Christensen H, Jorm AF (2008). Predictors of depression stigma. BMC Psychiatry.

[32] Ten Have M, de Graaf R, Ormel J, Vilagut G, Kovess V, Alonso J (2010). Are attitudes towards mental health help-seeking associated with service use? Results from the European Study of Epidemiology of Mental Disorders. Soc Psychiat Epidemiol.

[33] Ministry of Health Labour and Welfare (2015) Annual Health, Labour and Welfare Report 2014. Ministry of Health, Labour and Welfare. 2015. http://www.mhlw.go.jp/wp/hakusyo/kousei/15/dl/2-03.pdf. Accessed 25 Aug 2016 (in Japanese).

[34] Toth K, Dewa C (2014). Employee decision-making about disclosure of a mental disorder at work. J Occup Rehabil.

[35] Dewa C (2014). Worker attitudes towards mental health problems and disclosure. Int J Occup Environ Med.

[36] Brohan E, Thornicroft G (2010). Stigma and discrimination of mental health problems: workplace implications. Occup Med (Lond).

[37] Corrigan PW, River LP, Lundin RK (2001). Three strategies for changing attributions about severe mental illness. Schizophr Bull.

[38] Ministry of Health Labour and Welfare. Survey on state of employees’ health 2012. Ministry of Health, Labour and Welfare. 2013. http://www.mhlw.go.jp/toukei/list/h24-46-50.html. Accessed 25 Aug 2016 (in Japanese).

[39] Hasegawa T, Murata C, Ninomiya T (2013). Occupational factors and problem drinking among a Japanese working population. Ind Health.

[40] Kontos EZ, Emmons KM, Puleo E, Viswanath K (2012). Contribution of communication inequalities to disparities in human papillomavirus vaccine awareness and knowledge. Am J Public Health.

